# Genes implicated in multiple sclerosis pathogenesis from consilience of genotyping and expression profiles in relapse and remission

**DOI:** 10.1186/1471-2350-9-17

**Published:** 2008-03-19

**Authors:** Ariel T Arthur, Patricia J Armati, Chris Bye, Southern MS Genetics Consortium, Robert NS Heard, Graeme J Stewart, John D Pollard, David R Booth

**Affiliations:** 1 Department of Medicine and the Nerve Research Foundation, the University of Sydney, Sydney, Australia; 2 Institute for Immunology and Allergy Research, Westmead Millennium Institute, Sydney, Australia; 3 Westmead Millenium Institute, Sydney, Australia; 4 The University of Melbourne, Melbourne, Australia

## Abstract

**Background:**

Multiple sclerosis (MS) is an inflammatory demyelinating disease of the central nervous system (CNS). Although the pathogenesis of MS remains unknown, it is widely regarded as an autoimmune disease mediated by T-lymphocytes directed against myelin proteins and/or other oligodendrocyte epitopes.

**Methods:**

In this study we investigated the gene expression profiles of peripheral blood cells from patients with RRMS during the relapse and the remission phases utilizing gene microarray technology. Dysregulated genes encoded in regions associated with MS susceptibility from genomic screens or previous trancriptomic studies were identified. The proximal promoter region polymorphisms of two genes were tested for association with disease and expression level.

**Results:**

Distinct sets of dysregulated genes during the relapse and remission phases were identified including genes involved in apoptosis and inflammation. Three of these dysregulated genes have been previously implicated with MS susceptibility in genomic screens: TGFβ1, CD58 and DBC1. TGFβ1 has one common SNP in the proximal promoter: -508 T>C (rs1800469). Genotyping two Australian trio sets (total 620 families) found a trend for over-transmission of the T allele in MS in females (p < 0.13). Upregulation of CD58 and DBC1 in remission is consistent with their putative roles in promoting regulatory T cells and reducing cell proliferation, respectively. A fourth gene, ALOX5, is consistently found over-expressed in MS. Two common genetic variants were confirmed in the ALOX5 putatve promoter: -557 T>C (rs12762303) and a 6 bp tandem repeat polymorphism (GGGCGG) between position -147 and -176; but no evidence for transmission distortion found.

**Conclusion:**

The dysregulation of these genes tags their metabolic pathways for further investigation for potential therapeutic intervention.

## Background

MS the most common cause of chronic neurological disability in young adults with a lifetime risk of one in 400 in northern Europeans [1]. Most patients have the relapsing-remitting (RR) form of MS characterised by episodes of neurologic impairment followed by complete or almost complete recovery [2]. A relapse is thought to be caused by some trigger of the immune system resulting in the trafficking of activated, myelin-reactive T-cells into the CNS through a disrupted blood brain barrier (BBB) causing acute inflammation. Early clinical symptoms result from oligodendrocytic damage and/or demyelination. The regression of symptoms is attributed to resolution of immune-based attack and associated oedema and to partial remyelination or redistribution of sodium channels along demyelinated segments of axons [3]. Relapses may be mild or severe with exacerbations lasting several days to several weeks, or even months.

We aimed to investigate the gene expression profiles of whole blood from RRMS patients during the relapse and the remission phases utilizing gene microarrays and RT-PCR for confirmation of results. Whilst obtaining RNA samples for microarray analysis from brain tissue is problematic, peripheral blood is an appropriate tissue to study since it is the trafficking compartment of cells of the immune system and provides insight into the specific components that may be driving the immune response, contributing to both the disease and its exacerbations in RRMS. We used this transcriptomic information to find dysregulated genes, and then combined this with genetic association data linking genomic regions with MS, to find dysregulated genes encoded in genomic regions associated with MS. Since genomic studies have confirmed that genes are often regulated by their 5' untranslated regions [4,5], we then sought polymorphisms in these regions and tested them for association with MS.

We used four key approaches in our study. First, since demyelination is probably due largely to leukocytes in the peripheral circulation, and much of the variation in leukocyte expression is genetically controlled; we investigated the mRNA profiles of peripheral blood cells using whole blood rather than just mononuclear cells. Secondly, since many mRNA species are short-lived and subject to nucleases, blood was collected directly into a solution which immediately halts transcription and mRNA degradation. Thirdly, we recruited patients in remission who were not receiving immunomodulatory treatments and who had experienced 1–2 relapses within 2 years prior to blood sampling to ensure a patient cohort with a similar disease course and severity. Patients undergoing relapse were sampled on admission to hospital and prior to receiving any steroid treatment for their relapse. Finally, because aberrant gene expression may be an effect of MS rather than a cause, we investigated genetic differences in gene promoter regions, which would suggest inherited factors as a cause for the gene dysregulation.

Studies on gene expression signatures in MS from cells have indicated upregulation of pro-inflammatory, anti-apoptotic pathways [6]. Earlier, we found evidence of differences in gene expression between the progressive forms of the disease, primary progressive (PPMS) and secondary progressive MS [7], and by using the transcriptomic/genomic approach identified a CD127 haplotype associated with PPMS. The association of this gene with MS has now been widely replicated [8-10]. Here we use the same approach, and so identify TGFβ1, CD58 and DBC1 as implicated in MS pathogenesis. These and other identified dysregulated genes suggest the involvement of genes regulating the immune system and apoptosis in MS. Further, one gene in particular, ALOX5, is upregulated in previous studies [7,11], and in both our relapse and remission cohorts.

## Methods

### Patient and control blood samples

#### Gene expression

Blood samples from RRMS patients were collected at the MS Society Australia Clinic, or upon admittance to the Institute of Clinical Neuroscience, at Royal Prince Alfred Hospital, Sydney, NSW Australia. All patients recruited for this study were assessed independently by a neurologist who diagnosed a clinical relapse, in the case of the relapsing cohort, or confirmed clinical remission of the patients in the remission cohort. Blood samples were taken from relapsing patients prior to immunomodulatory treatment, whilst patients in remission were not receiving treatment and had experienced 1–2 relapses in the 2 years prior to blood sampling. Relapsing patients were between 30–66 years of age with a male to female ratio of 5:5, remission patients were between 30–55 years of age with a male to female ratio of 4:6. Control human blood samples were obtained from staff at Westmead Hospital, NSW Australia. The control individuals were Caucasian and between 20–55 years of age with a male to female ratio of 5:20. Female control RNA samples were pooled to obtain a single control sample. All blood samples (15 mls total) were numerically coded and obtained after informed consent according to the University of Sydney Human Ethics (protocol number 02/03/17) and the Central Sydney Area Health Service (CSAHS) Ethics (protocol number X02-0328).

#### DNA sequencing

Genomic DNA pools used for promoter region sequencing were constructed by the Institute for Immunology and Allergy Research. Genomic DNA samples from 217 HLA-DR2 positive MS patients, 155 HLA-DR2 negative MS patients, 169 probands from MS multiplex families, and from 187 unrelated healthy controls were pooled separately using the Ban et al protocol [12], based on previously described methods [13].

#### Genotyping

Trio families (MS patient, mother and father) used for genotyping were recruited by the Institute for Imunology and Allergy Research.

### RNA extraction

All whole blood samples were collected under standard phlebotomy protocol into PAXgene tubes (PreAnalytix, Switzerland) and RNA extracted from the samples using the PAXgene Blood RNA Kit (PreAnalytix, Switzerland). All methods followed the protocols supplied in the kit [14].

### RNA preparation for microarray analysis

The MessageAmp II Kit [15] was used to amplify the RNA for use with 10.5 K Peter MacCallum microarrays. These comprise ~10,500 elements representing 9,381 unique cDNAs (Unigene Build 144) and were printed onto superamine slides (Telechem) using a robotic arrayer (Virtek) by The Peter MacCallum Cancer Institute, Australia – full gene list published on the author's website [16]. The SuperScript Indirect cDNA Labelling System (Invitrogen) was used for cDNA production and CyDye (Amersham Biosciences) labeling. The protocol supplied was followed [17]. The labeled cDNA was hybridized at 65°C overnight, the arrays were washed and scanned using a 4000B scanner (AXON Instruments), then visualized using GenePix Pro 5.0 software. Each microarray was repeated using alternate CyDye combinations so that there were 2 arrays per patient. Each MS sample was compared to the female reference (pool of 20 females). Male controls were compared to this same reference. Genes different between the male controls and female reference were considered 'sex-specific' and excluded from the analysis of MS samples versus the reference sample.

### Microarray data analysis

Raw data files generated by the GenePix Pro software were loaded into the program R [18] and normalized using the freely available microarray analysis package Limma [18]. The data was normalized using the print-tip loess method and all spots with an intensity value less than 100 were filtered out of the data sets as these were considered unreliable. To avoid type 2 errors (false rejection of association) we used the Significance Analysis of Microarrays (SAM) package [19], which uses modified t tests to compare results for each gene and uses an iterative procedure to evaluate the likelihood of the association occurring by chance. The SAM package was used in conjunction with Limma to identify the most statistically significant dysregulated genes. The GOstat program [20] was used to identify gene ontology groups which were highly represented in the dysregulated gene sets.

### Quantitative real-time PCR

All primers were designed using Primer3 [21]. ALOX5 (H51574) forward primer sequence: 5'-CCACGGGGACTACATCGAGTT-3' and reverse primer sequence: 5'-CTTTACGTCGGTGTTGCTTGAG-3'. TGFβ1 (R36467) forward primer sequence: 5'-CGAGCCTGAGGCCGACTACTA-3' and reverse primer sequence: 5'-CTCGGAGCTCTGATGTGTTGAA-3'.

All RNA samples were quantified using RiboGreen reagent (Molecular Probes, Eugene, OR, USA) and fluorescence measured using a FluoroCount fluorescence microplate reader (Packard Bioscience, Meriden, CT, USA). Patient and control pool RNA was reverse-transcribed into cDNA by preparing 20 μl reverse transcription reaction containing: 500 ng RNA, 250 μg Oligo dT (Promega, Madison, WI, USA), 10 mM dNTPs (Promega, Madison, WI, USA). The reaction was incubated at 65°C for 5 min and cooled on ice, then 5× buffer (Promega, Madison, WI, USA), 40 U RNase inhibitor (Promega, Madison, WI, USA), and 400 U reverse transcriptase (Promega, Madison, WI, USA) was added and the reaction incubated at 42°C for 60 min then 80°C for 15 min.

SYBR Green (Applied Biosystems Foster City, CA, USA) was used to quantify cDNA by real-time PCR. The PCR reaction contained cDNA derived from 5 ng of MS or control total RNA, SYBR Green master mix (Applied Biosystems Foster City, CA, USA), and 100 ng of each primer. The reaction was amplified in a Corbett Rotor-Gene 2000 real-time PCR cycler (Corbett Research, NSW, Australia) using the following cycling profile: 1 cycle of 95°C for 10 min; 5 cycles (95°C for 30s, 64°C for 30s, 72°C for 30s); 35 cycles (95°C for 30s, 60°C for 30s, 72°C for 30s); then 72°C for 5 min. Melt curve analysis was then performed between 75–99°C in 1°C increments. RT-PCR products were electrophoresed on 2% 1× TBE agarose gels, stained with ethidium bromide (0.5 μg/ml) to visualize the product. Normalization to total RNA, or the 2^-ΔCt ^method [22] was used to determine differences in expression between the MS individuals and the control pool.

### Promoter region sequencing

Genomic DNA pools consisting of DNA samples from 217 HLA-DR2 positive MS patients, 155 HLA-DR2 negative MS patients, 169 probands from MS multiplex families, and from 187 unrelated healthy controls were used for promoter region sequencing. PCR primers were designed to amplify the 5' untranslated promoter region (approximately 500 bp upstream of the 1^st ^exon) using Primer3 [20]. ALOX5 (H51574) forward primer sequence: 5'-AGCCTCTGTGCTCCAGAATC-3' and reverse primer sequence: 5'-GGCTGAGGTAGATGTAGTCGTCA-3'. TGFβ1 (R36467) forward primer sequence: 5'-GGGAGGTGCTCAGTAAAGG-3' and reverse primer sequence: 5'-CTCGCTGTCTGGCTGCTC-3'. PCR reactions were prepared using 2× PCR Mastermix (MBI Fermentas, Vilinus, Lithuania), 100 ng of each PCR primer and 50 ng of pooled DNA samples or 0.1–1 μg of control individual genomic DNA. Reactions were amplified in the Mastercycler thermocycler (Eppendorf AG, Hamburg, Germany) using the following 64° -60°C "touchdown" temperature profile: 1 cycle of 95°C for 2 min 30 s; then 5 cycles (95°C for 30 s, 64°C for 30 s, 72°C for 30 s), then 30 cycles of (95°C for 30 s, 60°C for 30 s, 72°C for 30 s); then 1 cycle of 72°C for 5 min. For GC-rich sequences in the ALOX5 and TGFβ1 promoter regions, 5% DMSO and 0.02 U/μl Phusion DNA polymerase (Finnzymes, Finland) was also added to the reaction and a 62° -58°C "touchdown" temperature profile with a 98°C denaturation temperature, was used: 1 cycle of 98°C for 2 min 30 s; then 5 cycles (98°C for 30 s, 62°C for 30 s, 72°C for 30 s), then 30 cycles of (98°C for 30 s, 58°C for 30 s, 72°C for 30 s); then 1 cycle of 72°C for 5 min. PCR products were electrophoresed on 2% 1 × TBE agarose gels, stained with ethidium bromide (0.5 μg/ml) to visualize PCR products. The PCR products were then purified using 1 μl Exo-SAP-IT (exonuclease I/Shrimp alkaline phosphatase) (USB Corporation, Cleveland, OH, USA) incubated at 37°C for 15 min followed by 80°C for 15 min.

Unidirectional DNA sequencing was performed using an ABI Prism Big Dye terminator sequencing kit version 3.0 (Applied Biosystems, Foster City, CA, USA). PCR products were either sequenced in the forward or reverse direction using an internal primer. ALOX5 (H51574) reverse internal primer sequence: 5'-CATCTAGCGCCGCAGC-3'. TGFβ1 (R36467) forward internal primer sequence: 5'-CTCAGTAAAGGAGAGCAATTC-3'. Reactions were incubated at 95°C for 5 min, followed by 25 cycles (95°C for 30 s, 50°C for 15 s and 60°C for 4 min). Reactions were then electrophoresed on an ABI PRISM 3100 Genetic Analyzer (Applied Biosystems, Foster City, CA, USA) by the Westmead Millenium Institute DNA Sequencing Facility. DNA sequences were viewed using ABI Prism Editview software (Applied Biosystems, Foster City, CA, USA).

### Genotyping

Up to 388 trio families, recruited by the Institute for Immunology and Allergy Research, were utilized for this genotyping study. Since appropriate matching of controls is essential to avoid false positive associations resulting from population stratification or non-random mating, genetically unrelated family members of the MS patients are usually used as controls. However, confounding due to hidden stratification can still occur. For this reason we have used trio families in this study. When a patient and both parents (a "trio family") are considered, the non-transmitted parental alleles form the controls. In these trio families association was tested using the transmission disequilibrium test (TDT) [23].

One hundred and two trio families were genotyped for the ALOX5 6 bp (GGGCGG) deletion. PCR primers were designed to amplify the transcription factor binding region, containing the repeated motif GGGCGG, using Primer3 [21]. Forward primer: 5'-AGGTCCCGCCCAGTC-3' and reverse: 5'GGTCTGGCTCCAGGCT-3'. PCR reactions were prepared using 2× ImmoMix PCR Mastermix (Bioline), 100 ng of each PCR primer, 0.25 μl of 100% DMSO and 50 ng of DNA samples. Reactions were amplified in the Mastercycler thermocycler (Eppendorf AG, Hamburg, Germany) using the following 68° -64°C "touchdown" temperature profile: 1 cycle of 96°C for 10 min; then 5 cycles (96°C for 30 s, 68°C for 30 s, 72°C for 30 s), then 25 cycles of (96°C for 30 s, 64°C for 30 s, 72°C for 30 s); then 1 cycle of 72°C for 5 min. PCR products were electrophoresed on 15% acrylamide gels made with: 40% acrylamide; 10× TBE buffer; ammonium persulfate (100 mg/ml); TEMED; and H_2_O. The gels were stained with ethidium bromide (0.5 μg/ml) to visualize PCR products. Transmission disequilibrium tests (TDT) were performed using the Haploview program [24].

Three hundred and eighty eight trio families were genotyped for the TGFβ1 -508 C>T SNP. Genotyping of the TGFβ1 SNP was performed by the Australian Genome Research Facility (AGRF) (Queensland, Australia). The AGRF custom SNP genotyping service utilized the homogenous MassExtend (hME – single base extension) analysed on the Sequenom Autoflex Mass spectrometer and the Samsung 24 pin nanodispensor. SNP assays were designed by AGRF, PCR oligos were obtained and then processed in multiplex format, following PCR, on a mass spectrometer. The mass spectrometer allowed different SNPs to be identified with a high level of sensitivity. Transmission disequilibrium tests (TDT) were performed as above. The same genotyping strategy was employed for an independent cohort of 250 trios collected by Victorian institutions [25].

## Results

### Gene expression in RRMS: microarray vs. RT-PCR

#### Relapse

**Figure 1:**
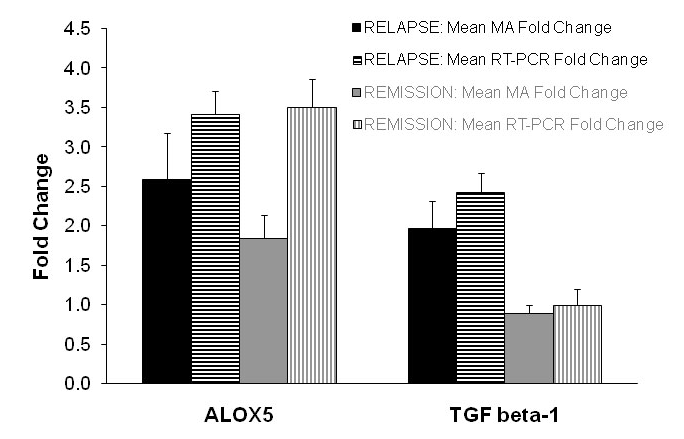
**Microarray vs RT-PCR results for ALOX5 and TGFβ1**. ALOX5 was up-regulated during relapse and remission while TGFβ1 was up-regulated only during the relapse phase.

The gene expression profile of 10 RRMS patients in clinical relapse compared to the control sample identified 989 statistically significant (p ≤ 0.05) up-regulated genes. Of particular interest, ALOX5 was up-regulated 2.58 (± 0.59) fold (mean microarray fold change for each individual), whilst the RT-PCR results showed a 3.41 (± 0.29) fold increase (Figure 1). TGFβ1 was up-regulated by 1.97 (± 0.34) according to the mean microarray results and 2.42 (± 0.24) according to RT-PCR (Figure 1). In addition, 536 statistically significant (p ≤ 0.05) genes were down-regulated. The complete dysregulated gene list (obtained from the limma analysis) for the relapse phase can be viewed on the author's website [16].

#### Remission

The gene expression profile of 10 RRMS patients in remission compared to the control sample identified 655 statistically significant (p ≤ 0.05) up-regulated genes. ALOX5 was also up-regulated during remission with a mean microarray fold change of 1.84 (± 0.29) and 3.51 (± 0.36) according to RT-PCR (Figure 1). According to the microarray and RT-PCR analysis, TGFβ1 was not up-regulated in RRMS patients during the remission phase. In addition, 662 statistically significant (p ≤ 0.05) genes were down-regulated. The complete dysregulated gene list (obtained from the limma analysis) for the remission phase can be viewed on the author's website [16]. It should be noted that due to the use of whole blood, fold change values of significant differentially expressed genes were relatively small, most around a value of 1.5–2.5.

### GO pathways over-represented in the dysregulated gene sets

Table 1 lists the statistically significant (p ≤ 0.05) over-represented pathways, in the relapse and remission upregulated gene sets, according to the GOstat program. There were no over- or under-represented pathways in the downregulated gene sets for either group. The over-represented pathways in the relapse upregulated gene set were predominantly inflammation/apoptosis-related, whilst the pathways in the remission upregulated gene set were quite distinct, being related instead to protein transport and localization.

**Table 1:** GO pathways over-represented in the relapse and remission upregulated gene sets.

**Disease Phase/Expression**	**Gene Ontology**	**p-value**	**Genes**
**RELAPSE Upregulated**	GO:0009607 – Response to biotic stimulus	0.0462	GPR65, NMI, TAP2, IFI16, CSF2RB, TCRIM, ICOS, FCGR2B, CD48, BST1, TNFSF10, TLR5, MCP, DNAJB6, MNDA, GBP2, OXSR1, AQP9, TNFAIP6, NFIL3, CD53, GEM, LY96, DOCK2, IL1B, CLECSF6, PARP4, PLA2G7, CD97, MX2, 5233, HSPA1B, MAPK14, IL1R2, C3AR1, IFIT2, STAT1, IFNGR1, S100A8, EDEM1, LYZ, FAS, HSPA1L, LTBR, IL1RL1, B2M, HSPA1A
	GO:0008219 – Cell death	0.0462	PARP4, GADD45B, MCL1, BCL2, GPR65, SMNDC1, RAF1, BIRC2, RAD21, CASP3, CASP10, TXNL1, BCLAF1, API5, STAT1, CUL1, YWHAG, TNFSF10, GADD45G, LYZ, FAS, IL1B, TRAF5, LTBR, TAX1BP1, CASP1, BID
	GO:0006955 – Immune response	0.0462	GPR65, NMI, TAP2, IFI16, CSF2RB, TCRIM, ICOS, FCGR2B, BST1, TNFSF10, TLR5, MCP, MNDA, GBP2, AQP9, NFIL3, TNFAIP6, CD53, GEM, DOCK2, LY96, IL1B, CLECSF6, PARP4, PLA2G7, CD97, MX2, MAPK14, IL1R2, C3AR1, IFIT2, S100A8, FAS, LTBR, IL1RL1, B2M
	GO:0016265 – Death	0.0462	PARP4, GADD45B, MCL1, BCL2, GPR65, SMNDC1, RAF1, BIRC2, RAD21, CASP3, CASP10, TXNL1, BCLAF1, API5, STAT1, CUL1, YWHAG, TNFSF10, GADD45G, LYZ, FAS, IL1B, TRAF5, LTBR, TAX1BP1, CASP1, BID
	GO:0006952 – Defense response	0.0462	GPR65, NMI, TAP2, IFI16, CSF2RB, TCRIM, ICOS, FCGR2B, CD48, BST1, TNFSF10, TLR5, MCP, MNDA, GBP2, AQP9, NFIL3, TNFAIP6, CD53, GEM, DOCK2, LY96, IL1B, CLECSF6, PARP4, PLA2G7, CD97, MX2, MAPK14, IL1R2, C3AR1, IFIT2, S100A8, LYZ, FAS, LTBR, IL1RL1, B2M
	GO:0004722 – Protein serine/threonine phosphatase activity	0.0462	MTMR6, PPM1A, PPM1B, DUSP6, PPP2R2A, MTMR1
	GO:0006915 – Apoptosis	0.0462	GADD45B, MCL1, BCL2, GPR65, SMNDC1, RAF1, BIRC2, RAD21, CASP3, CASP10, TXNL1, BCLAF1, API5, STAT1, YWHAG, CUL1, TNFSF10, GADD45G, FAS, IL1B, TRAF5, LTBR, TAX1BP1, CASP1, BID
	GO:0012501 – Programmed cell death	0.0462	GADD45B, MCL1, BCL2, GPR65, SMNDC1, RAF1, BIRC2, RAD21, CASP3, CASP10, TXNL1, BCLAF1, API5, STAT1, YWHAG, CUL1, TNFSF10, GADD45G, FAS, IL1B, TRAF5, LTBR, TAX1BP1, CASP1, BID
	GO:0005057 – Receptor signaling protein activity	0.0533	STAT1, TGFBR2, RAF1, LYN, ACVR1B, MAPK14, NSMAF, MAPK9, IRS1, TCRIM, IL1RL1, PDLIM5, MAPK8
**REMISSION Upregulated**	GO:0015031- protein transport	0.0189	STXBP3, RANBP6, RAB2, PTTG1IP, ARF1, STX7, SNX2, GRP58, TLOC1, KPNB1, VDP, SNAG1, CLTC, COPB, TMED7, ARF3, RAB5A, TNPO1, RAB1A, COPA, RAB7, TLK1
	GO:0045184 – establishment of protein localization	0.0189	STXBP3, RANBP6, RAB2, PTTG1IP, ARF1, STX7, SNX2, GRP58, TLOC1, KPNB1, VDP, SNAG1, CLTC, COPB, TMED7, ARF3, RAB5A, TNPO1, RAB1A, COPA, RAB7, TLK1
	GO:0008104 – protein localization	0.0189	STXBP3, RANBP6, RAB2, PTTG1IP, ARF1, STX7, SNX2, GRP58, TLOC1, KPNB1, VDP, SNAG1, CLTC, COPB, TMED7, ARF3, RAB5A, TNPO1, RAB1A, COPA, RAB7, TLK1
	GO:0005885 – Arp2/3 protein complex	0.046	ARPC2, ACTR3, ARPC3
	GO:0030833 – regulation of actin filament polymerization	0.046	ARPC5, ARPC2, ARPC3

### Screening dysregulated genes for genomic association with MS

Prior to 2007, the two largest genomic screens have been that of the GAMES collaborative using > 4000 microsatellite markers in a case controls study [26], and the affected family study using 400 SNPs in 1000 individuals [27]. The latter identified 5q33, 17q23 and 19p13 as having the highest LOD scores of the non-HLA region, and several genes from the most dysregulated set in relapse and remission were encoded in these genomic regions (Table 2). The meta-analysis from the GAMES study found 3 microsatellite markers associated with MS, D11S1986, D19S552, and D20S894. Only 3 genes were encoded within 1 MB, the likely maximum linkage distance, of any of these markers: TGFβ1, CEACAM1 and the glial maturation factor (all from 19q13.2). Only TGFβ1 is immune cell specific [28], so this gene was selected for further study. A more recent screen by the international multiple sclerosis consortium (IMSGC), has provided a more comprehensive coverage of the genome [10], and identified 38 genes with primary evidence for association. Of these 4 were dysregulated in our study: CD58, DBC1, TAF1A, and FHIT (Table 2). The genetic association of CD58 and DBC1 were confirmed in the IMSGC's replication study.

**Table 2:** Dysregulated genes from patients in relapse and remission encoded in genomic regions associated with MS.

**Chromosomal Region Associated with MS**	**Genes Upregulated in Relapse**	**Genes Downregulated in Relapse**
5q33^1^	SOX30	0
17q23^1^	FTSJ3, USP32, MRC2, TLK2, PRKAR1A	SMARCD2, HAN11WD
19p13^1^	CD97, HOMER3, THRAP5, ILF3, OAZ1, AES, FUTC, SAFB, C19ORF6, ARHGEF18, SF4, GADD45B, CYP4F12	RGS19IP1, DHPS, MGC11271, KLF1, GCDH, FLJ90396, AZU1, FARSLA, KHSRP, TRIP10, ILVB1, ANGPTL4, NDUB7, NDUFA, MADCAM17
11q23 (D11S1986) ^2^	0	0
19q13.2 (D19S552) ^2^	TGFB1, CEACAM1, GMF	0
20p12 (D20S894) ^2^	0	0
IMSGC^3^	TAF1A	FHIT, **DBC1**

	**Genes Upregulated in Remission**	**Genes Downregulated in Remission**

5q33^1^	0	0
17q23^1^	0	0
19p13^1^	0	0
11q23 (D11S1986) ^2^	SDHD, CASP1, SIAT4C, APLP2	MGC2574, CD3E, RARRES3, CD3G, MTVR1, PCSK7
19q13.2 (D19S552) ^2^	CEACAM4, VASP	PSG6, HNRPUL1, PVR, EIF3S12
20p12 (D20S894) ^2^	0	0
IMSGC^3^	**CD58**	FHIT

Chromosomal regions are those identified as associated by Sawcer et al (2005)^1 ^[27] from the family study or by the GAMES collaboration (2006)^2 ^[26] using microsatellites (as marked) in a case/control study, and from the IMSGC SNP based genome wide analysis^3 ^[10]. Genes are from the set of genes dysregulated in relapse and remission. Highlighted genes have been associated with MS in replication cohorts.

### Genotyping of the TGFβ1 promoter region

In the TGFβ1 promoter sequence, one known SNP at position -508 T>C (rs1800469) was confirmed in both the MS and control populations (Table 3). No other previously identified or common novel SNPs were found. This SNP was not associated with MS overall in the Westmead cohort (380 trios), but as there was a trend (P < 0.13), and the statistical power may not have been sufficient, we increased the cohort size to 620 trios. However, in the combined cohort there was still no significant association.

**Table 3:** Genetic variants in the putative ALOX5 and TGFβ1 promoters.

**Gene**	**GenBank Accession no.**	**Common SNPs in the 5' upstream promoter region**	**SNPs found in this study**
Arachidonate 5- Lipoxygenase (ALOX5)	H51574	rs12762303: -557 T>C (Hoshiko et al, 1990) -458 T>C (ensembl database)	Both identified in MS and control cohorts
		GGGCGG tandem repeat deletion: Between -146 and -176 (Hoshiko et al,1990) Between -48 and -78 (ensembl database)	
Transforming growth factor beta-1 (TGFβ1)	R364674	rs12977628: -18 C>A (Kim et al, 1989) +181 C>A (ensembl database)	rs1800469 identified in MS and control cohorts
		rs17516265: -257 A>C (Kim et al, 1989) -59 A>C (ensembl database)	
		rs35318502: -315 C>T (Kim et al, 1989) -117 C>T (ensembl database)	
		rs35775330: -331 TT del (Kim et al, 1989) -133 TT del (ensembl database)	
		rs11466314: -448 G>A (Kim et al, 1989) -250 G>A (ensembl database)	
		rs1800469: -508 T>C (Kim et al, 1989) -310 T>C (ensembl database)	

Sequencing of the 5' upstream promoter region of pooled genomic DNA confirmed the presence of two common genetic variants in the ALOX5 promoter: rs12762303 T>C and a 6 bp deletion of the tandem repeat sequence GGGCGG, in addition to a single common SNP in the TGFβ1 promoter: rs1800469 T>C. Common SNPs and their positions in the 5' promoter region are shown according to nucleotide numbering in the ensembl database and by the authors who first described the promoter regions for ALOX5 (Hoshiko et al, 1990) [66] and TGFβ1 (Kim et al, 1989) [48]. Pooled DNA sequencing only detects common polymorphisms (> 10–15% of sample) [30].

### Genotyping of the ALOX5 promoter region

Because the gene association studies do not exclude regions as having associations, we examined the promoter region of ALOX5, which is upregulated in many MS peripheral blood gene expression arrays [7,11], including both the relapse and remission sets here. In the ALOX5 5'UTR sequence, one known SNP at position -557 T>C (rs12762303) was confirmed in both the MS and control populations, in addition to a deletion of one of the 6 bp tandem repeats (GGGCGG) of the consensus Sp1 binding sequence located between position -147 and -176 upstream from the ATG start site of the gene, as identified by In et al [29] (Table 3). This region also contains 5 Egr-1 binding motifs, located between position -148 and -180.

When 102 trio families were genotyped for the ALOX5 6 bp GGGCGG deletion, TDT testing showed no evidence of transmission distortion for the deletion with 38 long (normal sequence): 34 short (deletion) transmitted (p < 0.6). The short allele (minor allele) had a frequency of 0.19 in the MS population and 0.20 in the parental population. This deletion appears therefore not to be a significant risk factor in MS. We aimed to have the power to detect at least a 1.5- to 2-fold risk; however, a larger sample would be required for statistical power to detect a smaller risk factor. According to the McGinnis method, for trios, a 1.5-fold risk requires 266 families for an 80% chance of detecting association at a 5% significance level whilst a 2-fold risk requires 85 families. Due to the small sample size no stratification was performed. Both the -557 SNPs were equally represented in the control and MS (DR+, DR-, Familial) pools, and so was not examined further [30].

## Discussion

### Gene expression profiles of RRMS: relapse vs. remission

This study found a large number of dysregulated genes in peripheral blood during the relapse and remission phases of RRMS. When these sets of genes were compared against the total gene set contained on the array, there was a statistically significant over-representation of apoptosis- and inflammatory-related gene ontologies in the relapse up-regulated gene set. This was expected as MS relapse is associated with detrimental inflammation in which increases in inflammatory cell activation and the activation of inflammatory-related pathways is predominant. Recent studies have also found upregulation of inflammatory anti-apoptotic pathways in relapse [6], as a distinct MS cluster [31], or in MS compared to controls [32-34]. This is also consistent with the success of immunomodulatory therapies in RRMS [35]. In the remission up-regulated gene set, there was an over-representation of protein transport and localization, and actin polymerisation ontologies. Since these processes are altered when cells change shape and motility, they may indicate that fluctuation in cell trafficking is important in remission.

To date, microarray studies using MS blood have focused on peripheral blood mononuclear cells. Recently, the involvement of other blood cells in MS disease pathogenesis, such as neutrophils, has raised interest due to increasing evidence suggesting that elements of the immune response classically associated with allergy, may contribute to the pathogenesis of autoimmune CNS demyelinating diseases, such as MS and its animal model EAE [36]. We have identified the upregulation, both during relapse and remission, of arachidonate 5-lipoxygenase (ALOX5), an enzyme that catalyzes the initial steps in the conversion of arachidonic acid to biologically active leukotrienes in neutrophils [37,38]. Interestingly, we previously found an up-regulation of ALOX5 in both secondary progressive and primary progressive MS patients [7]. Gene expression studies of MS and EAE lesions have also reported increased levels of ALOX5 [11] in addition to other allergy-related mediators [39-41].

### Gene promoter variation and expression

Combining genomic and transcriptomic data has proved useful in identifying genetic associations in disease. In this way we earlier identified CD127 polymorphisms as associated with PPMS [7], an association now independently confirmed in two large cohorts [8,9]. The general principal of finding SNPs affecting expression in the 5' UTR region has also now been established by combining expression phenotype and HapMap data [4] or from the ENCODE project [5]. We have examined the putative promoter regions of TGFβ1 and ALOX5 for polymorphisms then tested the association of these variants with MS. A further advantage of this approach is that if promoter polymorphisms are associated with MS, then this provides support for the observed aberrant expression being pathogenic rather than homeostatic.

Genomic regions chosen for screening of dysregulated genes were based on the current largest genomic studies [10,26,27]. The genomic region from the affected family study was large, and this is reflected in the large number of genes selected using these criteria. Three genes were targeted from the more stringent criteria provided by the GAMES study [26], and TGFβ1 was selected for further study as it is the only one whose expression is confined to immune cells.

### Variations in the TGFβ1 promoter region

In addition to being close to the microsatellite in the GAMES meta analysis [26], in 19q13.2, in which TGFβ1 is encoded, this gene has been identified as being associated with MS susceptibility in other studies [42-47]. Dysregulated expression of TGFβ1 may have significant consequences in MS pathogenesis and potentially could be due to sequence variation in the TGFβ1 promoter region. Only one common SNP, -508 T>C, was detected in the 500 bp 5' UTR putative promoter sequence [48] (Table 3). From twin studies, Grainger et al [49] demonstrated TGFβ1 expression was under genetic control, and that the T allele is associated with higher expression. Two groups have studied the association of this SNP with MS [50,51]. We found evidence for an association when 102 trios were genotyped, and then increased the numbers to 388 trio families. This is well in excess of the number needed to have the statistical power to have an 80% chance of detecting a risk factor of 1.5 at the 0.05 significance level (212 families needed according to the McGinnis method). We identified a trend towards over-transmission of the T allele in MS in our patient cohort. We then tested the association in a second independent trio cohort, but found only non-significant over-transmission (Table 4).

**Table 4:** Genotyping of the TGFβ1 -508 T>C SNP (rs1800469) in 423 informative MS trio families.

**Group**	**T:C**	**P-value**
Westmead	139T:115C	0.13
SMSGC	88T:81C	ns
All (both cohorts)	227T:196C	0.13
IMSGC^[10]^	C>T (1.1 odds ratio)	0.20

The samples were stratified and both corrected and uncorrected p-values were calculated to avoid type I and type II errors. There is a trend for over-transmission of the T allele in MS (p < 0.13), and an association in females (p_uncorrected_<0.04), HLA-DR+ (p_uncorrected_<0.01), which was also found when data was combined from a second independent cohort (SMSGC), but was not significant after correction for multiple testing. However, the IMSGC transmission was in the reverse order, with C overtransmitted [10]. This was inferred from the transmission of rs2241714, which is in LD with rs1800469.

Interestingly Green et al, in contrast to Weinshenker et al, found the wild-type C allele (or the "low-producer" genotype), which was under-transmitted in our study, was associated with a milder disease course. EAE and human drug response data suggest high TGFβ1 expression would be protective [52-56]. Green et al [50] also found strong linkage disequilibrium (LD) existing between the SNP at position -508 and the codon 10 SNP and partial LD with two other codon changing SNPs, which may result in an altered function of the translated protein, which may in turn be the cause of the association with susceptibility found in our study. Neither Green et al (2001), nor Weinshenker et al (2001), found an association between the -508 SNP and MS susceptibility (Table 5). A final assessment of the significance of TGFβ1 as a risk factor will be possible after larger, multiple cohorts have been studied, with informed stratification for clinical parameters, and after testing of epistatic interactions.

TGFβ1 is a multifunctional growth factor with demonstrable immunosuppressive effects on a number of cells including B cells, CD4+ T cells (Th1 and Th2), CD8+ cytotoxic T lymphocytes, natural killer cells and macrophages [57] and has been recently been implicated as a key factor in the differentiation of Th17 and T regulatory cells [58]. The full significance of TGFβ1-mediated effects on immune responses is still incompletely understood, but have been implicated in MS pathogenesis, with some studies finding reduced TGFβ1 levels during a relapse and an increase during remission [59-62]. However, it has also been reported that TGFβ1 levels increase during remission and even more so during relapse and in progressive patients [63].

**Table 5:** T allele frequencies of the TGFβ1 -508 T>C SNP (rs1800469) as found by this study (*) and other studies.

**T Allele Frequency**		**Population Studied**	**Reference**
		
**Disease**	**Control**		
-	0.31	Healthy female twins	Grainger et al. (1999) ^[49]^
0.33	0.31	Prostate cancer	Ewart-Toland et al. (2004) ^[67]^
Belgian: 0.32 Canadian: 0.32	Belgian: 0.31 Canadian: 0.32	Abdominal aortic aneurysms	Ogata et al. (2005) ^[68]^
All IBD: 0.34 Crohn's disease: 0.42 Ulcerative colitis: 0.35	0.26	Inflammatory bowel disease (IBD) – Crohn's disease and ulcerative colitis	Schulte et al. (2001) ^[69]^
0.28 ^#^	0.29 ^#^	Multiple sclerosis	Green et al. (2001) ^[50]^
0.29	0.27	Multiple sclerosis	Weinshenker et al. (2001) ^[51]^
0.33	0.30	Multiple sclerosis*	This study

^# ^Green et al [50] investigated the frequencies of TGFβ1 haplotypes comprising of five biallelic polymorphisms, including two in the promoter region (one being the -508 T>C) and three in coding regions. Three haplotypes: GTCGC; GTTGC; and GTCGC, each containing the T allele (bold) at position -508 had frequencies of 0.24, 0.025 and 0.018 (0.28) in the controls and 0.25, 0.019, and 0.018 (0.29) in the MS individuals with no significant difference between the two groups.

### Variations in the ALOX5 promoter region

This study is the first to investigate the ALOX5 promoter variations in MS patients. We did not identify any association between the variant 5'UTR alleles and MS, suggesting that these insertion/deletion (in/del) variants in the Sp1/Egr-1 binding sites are not pathogenic in MS, and may not be the cause of the increased ALOX5 expression measured in MS patients by microarray analysis. However, extensive quantitative studies would be needed to confirm this. As mentioned previously, our small sample size does not exclude this in/del as an appreciable risk factor. It is possible that in MS, as found in asthma [64], these promoter variations may affect disease modification rather than disease susceptibility – an idea worthy of further investigation if ALOX5 inhibitors were considered for MS treatment.

### IMSGC whole genome analysis: genes in the dysregulated set

Of the 38 genes implicated in MS susceptibility on the first screen in the recently published whole genome screen, four were in the dysregulated gene sets identified here (Table 2). Two of these four genes were further supported as associated with MS susceptibility in their replication study: CD58 and DBC1. CD58, in common with the most associated genes from that and other studies [8-10], IL7Rα and IL2Rα, affects regulatory T cell (Treg) differentiation and proliferation. TGFβ1 is essential for Treg differentiation. The up-regulation of CD58 in remission is consistent with the evidence that the protective allele is associated with higher CD58 expression, and that CD58 promotes Treg differentiation. Gene expression and genotyping data therefore further implicate Tregs in MS pathogenesis. Notably, the two most associated genes from the IMSGC study, IL7Rα and IL2Rα, were not detected as dysregulated in whole blood. DBC1 is anti-proliferative [65], so that the higher expression observed in remission is consistent with an immunosuppressive function.

## Conclusion

We have reported the gene expression profiles of RRMS during the relapse and remission phases, identifying numerous dysregulated genes which may be specific to RRMS pathogenesis. Up-regulation of ALOX5 during relapse and remission and in progressive disease [7], suggests a role for this molecule in MS. Higher expression of ALOX5 in whole blood supports a possible role for neutrophils and allergic component in MS pathogenesis. A pathogenic outcome of high expression could be increased permeability of the blood brain barrier. No association of ALOX5 promoter polymorphisms with MS susceptibility was detected, but a much larger sample size would be needed to exclude it as an appreciable risk factor.

The interpretation of the up-regulation of TGFβ1 and it role in MS pathogenesis, is challenging. TGFβ1 has a multitude of functions and there is evidence to support both an immunosuppressive and a proinflammatory role for it in MS pathogenesis. However, we propose that the observed up-regulation of TGFβ1 during relapse may be in response to the proinflammatory state and is acting to promote Treg cell proliferation. This is consistent with the observed increase in expression of CD58 in remission, a gene now with strong support for association with MS. Genotyping of the C>T SNP identified in the promoter region of the TGFβ1 gene, revealed non-significant over-transmission of the T allele in MS. Additional studies in independent and larger cohorts stratified for these parameters are needed. Further study is appealing, as confirmation of any genetic association and its phenotypic effect will define a pathogenic process and target for novel therapeutic interventions.

## Abbreviations

MS – multiple sclerosis

CNS – central nervous system

RRMS – relapsing remitting multiple sclerosis

PPMS – primary progressive multiple sclerosis

TGFβ1 – transforming growth factor beta-1

ALOX5 – arachidonate 5-lipoxygenase

CD58 – leukocyte function antigen 3

DBC-1 – deleted in bladder cancer 1

CD127 – interleukin 7 receptor

FHIT – fragile histidine triad gene

TAF1A – transcription factor 1A

SNP – single nucleotide polymorphism

BBB – blood brain barrier

RT-PCR – real-time polymerase chain reaction

TDT – transmission disequilibrium test

## Competing interests

The author(s) declare that they have no competing interests.

## Authors' contributions

ATA carried out the microarray, RT-PCR and sequencing studies. ATA and CB carried out microarray data analysis. DRB and SMSGC performed the genotyping. PJA, GJS and DRB conceived of the study, participated in its design and supervised the study. JDP and RNSH recruited, and collected blood from, MS patients for the study. ATA and DRB drafted the manuscript. All authors read and approved the final manuscript.

## Pre-publication history

The pre-publication history for this paper can be accessed here:


